# Therapeutic drug monitoring guided dosing versus standard dosing of alectinib in advanced ALK positive non-small cell lung cancer patients: Study protocol for an international, multicenter phase IV randomized controlled trial (ADAPT ALEC)

**DOI:** 10.3389/fonc.2023.1136221

**Published:** 2023-03-09

**Authors:** Marinda Meertens, M. Benthe Muntinghe-Wagenaar, Barend J. Sikkema, Marta Lopez-Yurda, Valesca P. Retèl, Marthe S. Paats, Rob Ter Heine, Ed Schuuring, Wim Timens, Daan J. Touw, Job F. M. van Boven, Adrianus. J. de Langen, Sayed M. S. Hashemi, Lizza E. L. Hendriks, Sander Croes, Michel M. van den Heuvel, Anne-Marie C. Dingemans, Ron H. J. Mathijssen, Egbert F. Smit, Alwin D. R. Huitema, Neeltje Steeghs, Anthonie J. van der Wekken

**Affiliations:** ^1^ Department of Pharmacy and Pharmacology, Netherlands Cancer Institute, Amsterdam, Netherlands; ^2^ Department of Pulmonology and Tuberculosis, University of Groningen, University Medical Center Groningen, Groningen, Netherlands; ^3^ Department of Medical Oncology, Erasmus MC Cancer Institute, University Medical Center Rotterdam, Rotterdam, Netherlands; ^4^ Department of Biometrics, The Netherlands Cancer Institute, Amsterdam, Netherlands; ^5^ Division of Psychosocial Research and Epidemiology, Netherlands Cancer Institute, Amsterdam, Netherlands; ^6^ Department of Pulmonary Medicine, Erasmus MC Cancer Institute, University Medical Center Rotterdam, Rotterdam, Netherlands; ^7^ Department of Pharmacy, Radboud Institute for Health Sciences, Radboud University Medical Center Nijmegen, Nijmegen, Netherlands; ^8^ Department of Pathology and Medical Biology, University Medical Center Groningen, University of Groningen, Groningen, Netherlands; ^9^ Department of Clinical Pharmacy and Pharmacology, University Medical Center Groningen, University of Groningen, Groningen, Netherlands; ^10^ Department of Thoracic Oncology, Netherlands Cancer Institute-Antoni van Leeuwenhoek Hospital, Amsterdam, Netherlands; ^11^ Department of Pulmonary Medicine, Amsterdam University Medical Center, VU University Medical Center, Amsterdam, Netherlands; ^12^ Department of Respiratory Medicine, Maastricht University Medical Center, GROW School for Oncology and Reproduction, Maastricht, Netherlands; ^13^ Department of Clinical Pharmacy and Toxicology, Maastricht University Medical Center, CARIM School for Cardiovascular disease, Maastricht, Netherlands; ^14^ Department of Pulmonary Diseases, Radboud University Medical Center, Nijmegen, Netherlands; ^15^ Department of Pulmonology, Leiden University Medical Center, Leiden, Netherlands; ^16^ Department of Clinical Pharmacy, University Medical Center Utrecht, Utrecht, Netherlands; ^17^ Department of Pharmacology, Princess Máxima Center for Pediatric Oncology, Utrecht, Netherlands; ^18^ Department of Clinical Pharmacology, Division of Medical Oncology, The Netherlands Cancer Institute, Antoni van Leeuwenhoek Hospital, Amsterdam, Netherlands

**Keywords:** non-small cell lung cancer, anaplastic lymphoma kinase, protein kinase inhibitors, therapeutic drug monitoring, randomized controlled trial, pharmacokinetics, personalized dosing, cost-effectiveness

## Abstract

**Background:**

Alectinib is first-line therapy in patients with stage IV non-small cell lung carcinoma (NSCLC) and an anaplastic lymphoma kinase (ALK) fusion. A shorter median progression-free survival (mPFS) was observed when alectinib minimum plasma concentrations during steady state (C_min,SS_) were below 435 ng/mL. This may suggest that patients should have an alectinib C_min,SS_ ≥ 435 ng/mL for a more favorable outcome. This potential target could be attained by using therapeutic drug monitoring (TDM), i.e. adjusting the dose based on measured plasma trough concentrations. Hypothetically, this will increase mPFS, but this has not yet been evaluated in a randomized controlled trial (RCT). Therefore, the ADAPT ALEC trial is designed, with the primary objective to prolong mPFS in NSCLC patients treated with alectinib by using TDM.

**Methods:**

ADAPT ALEC is a multicenter, phase IV RCT, in which patients aged ≥ 18 years with advanced ALK positive (+) NSCLC eligible for alectinib in daily care are enrolled. Participants will be randomized (1:1 ratio) into intervention arm A (TDM) or B (control), stratified by brain metastases and prior ALK treatments. Starting dose in both arms is the approved flat fixed dose of alectinib 600 mg taken twice daily with food. In case of alectinib C_min,SS_ < 435 ng/mL, arm A will receive increased doses of alectinib till C_min,SS _≥ 435 ng/mL when considered tolerable. The primary outcome is mPFS, where progressive disease is defined according to RECIST v1.1 or all-cause death and assessed by CT-scans and MRI brain. Secondary endpoints are feasibility and tolerability of TDM, patient and physician adherence, overall response rate, median overall survival, intracranial PFS, quality of life, toxicity, alectinib-M4 concentrations and cost-effectiveness of TDM. Exploratory endpoints are circulating tumor DNA and body composition.

**Discussion:**

The ADAPT ALEC will show whether treatment outcomes of patients with advanced ALK+ NSCLC improve when using TDM-guided dosing of alectinib instead of fixed dosing. The results will provide high quality evidence for deciding whether TDM should be implemented as standard of care and this will have important consequences for the prescribing of alectinib.

**Clinical trial registration:**

ClinicalTrials.gov, identifier NCT05525338.

## 1 Introduction

The identification of targetable oncogenic driver mutations and the introduction of tyrosine kinase inhibitors (TKIs) as targeted therapy in non-small cell lung cancer (NSCLC) improved the survival of NSCLC patients considerably (1–4). In the Netherlands, in 1.3% of the patients with advanced NSCLC an anaplastic lymphoma kinase (ALK) fusion is detected, which make these patients eligible for targeted therapy with an ALK TKI (5). Alectinib was registered in 2015 as second-line therapy in ALK positive (+) patients, after first-generation ALK TKI crizotinib (6). This changed after publication of the randomized phase III J-ALEX and ALEX trials, where alectinib showed improved median progression-free survival (mPFS) compared to crizotinib (7, 8). Ever since, alectinib is standard of care in first- and second-line setting in ALK+ NSCLC treatment (9, 10). In updated overall survival (OS) data of the ALEX trial, the median OS of alectinib was not reached, whereas the median OS of crizotinib was 57.4 months (stratified hazard ratio (HR) 0.67, P = 0.0367), median follow-up durations were 48.2 months and 23.3 months respectively (11). Therefore, alectinib showed meaningful improvement of the OS compared to crizotinib.

The approved standard dose of alectinib in Europe is a fixed dose of 600 mg twice daily (BID) (12). A retrospective exposure-response analysis of alectinib showed that mPFS was significantly longer in patients with an exposure above the minimum plasma concentration during steady state (C_min,SS_) of 435 ng/mL compared to patients with trough levels < 435 ng/mL (13). This suggests that patients with alectinib C_min,SS_ ≥ 435 ng/mL had a more favorable outcome. The median alectinib C_min,SS_ per patient was 517 ng/mL (range: 141-1944 ng/mL), with an interindividual variability of 57% (13). Available data support pharmacokinetic (PK) guided dose increases in patients with low exposure, with C_min,SS_ ≥ 435 ng/mL as a proposed target. This could be attained by using therapeutic drug monitoring (TDM), i.e. adjusting the dose based on measured plasma concentrations in the individual patient.

However, in another exposure-response analysis, no association was found between OS and trough levels of both alectinib and the active metabolite M4 (14). Therefore, the evidence for TDM-guided dosing of alectinib is conflicting, as results of two performed exposure-response analyses were not consistent with each other (13, 14). This stressed the high demand for prospective randomized controlled trials (RCTs) to ensure the level of evidence of TDM of TKIs including alectinib. Recently, a prospective multicenter study by the Dutch Pharmacology Oncology Group showed TDM-guided dosing of various oral targeted anti-cancer agents to be feasible in clinical practice, which resulted in a reduced proportion of patients with a C_min,SS_ below the proposed target. For alectinib only 14 evaluable patients were included, of which two received a successful dose intervention (15). Based on this data, it cannot yet be said whether TDM has a potential clinical benefit for alectinib. While the exposure-response analysis suggests a potential clinical benefit of TDM-guided dosing of alectinib, there is not enough support yet for its implementation in clinical practice. For this reason, there is a great demand to study the clinical benefit of TDM in an RCT.

In this paper, we present the outline of the ADAPT ALEC trial: a prospective, multicenter, phase IV, RCT to study the clinical benefit of TDM in patients with advanced ALK+ NSCLC. The obtained study results will address the question whether personalized dosing results in a significant difference in the treatment outcomes of individual patients with ALK+ NSCLC.

## 2 Methods and analysis

### 2.1 Aim and objectives

The aim of this RCT is to investigate the effect of TDM-guided dosing of alectinib on treatment outcomes of patients with ALK+ NSCLC. We hypothesize that using TDM to increase the dose of alectinib in patients with C_min,SS_ < 435 ng/mL if tolerable, will raise the mPFS. The primary objective will be to demonstrate, among patients who had a C_min,SS_ < 435 ng/mL at any time point during treatment, a prolonged mPFS in the TDM-guided dosing arm compared to the fixed dosing arm. Feasibility and tolerability of TDM, patient and physician adherence, overall response rate (ORR), median overall survival (mOS), intracranial PFS, quality of life (QoL), toxicity, alectinib-M4 concentrations and cost-effectiveness of TDM will be evaluated as secondary objectives. In addition, exploratory data will be collected on circulating tumor DNA (ctDNA) and its association with response and types of resistance to therapy. Data regarding the influence of alectinib on body composition will also be collected as exploratory objective, as weight gain is a known but not well understood side-effect.

### 2.2 Study design

**Figure 1:**
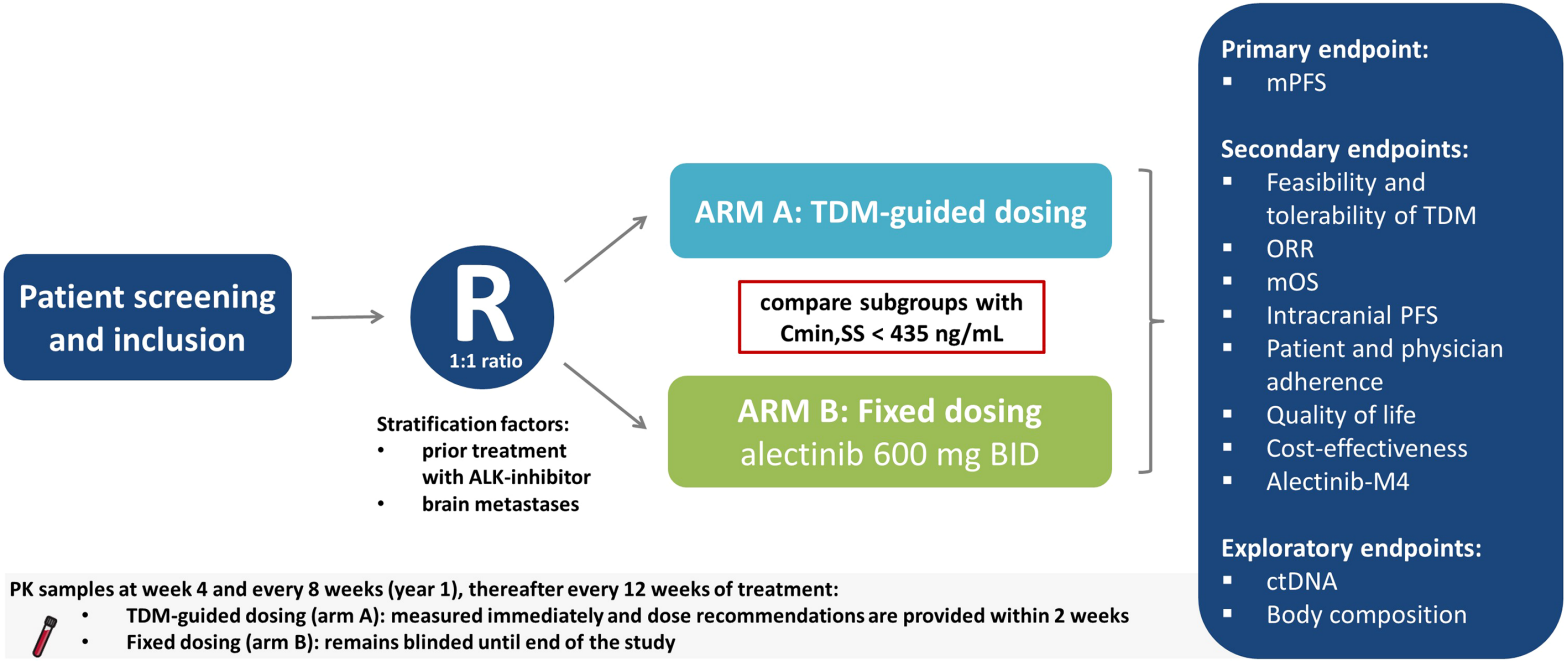
Schematic overview of study design. ALK, Anaplastic lymphoma kinase; BID, twice daily; C_min,SS_, minimum plasma concentration during steady state; ctDNA, circulating tumor DNA; mPFS, median progression free survival; mOS, median overall survival; ORR, overall response rate; R, Randomization; TDM, Therapeutic Drug Monitoring.

The ADAPT ALEC trial is an international, multicenter, phase IV, RCT comparing TDM-guided dosing to standard dosing of alectinib. Initially the trial will be open at seven study sites in the Netherlands, after which international sites will follow. The study design is displayed in **Figure 1**. Patients will be centrally randomized according to the minimization method in a 1:1 ratio, stratified by prior treatment with other ALK inhibitors and presence of brain metastases, using the online randomization tool ALEA v.18.3 (FormsVision BV, Abcoude, The Netherlands). The trial will compare, in the subgroup of patients with alectinib plasma concentration C_min,SS_ < 435 ng/mL at any time point, those receiving dose-adjusted alectinib based on TDM (arm A) with those receiving standard treatment with fixed dose alectinib (arm B).

Patients who start alectinib treatment at the approved European standard dose of 600 mg BID will be screened and if eligible, randomized into arm A or B. First PK samples will be collected at 4 weeks after start of treatment. Subsequently, plasma samples will be drawn to evaluate the plasma trough concentration every 8 weeks in the first year of treatment, then every 12 weeks thereafter. Blood collection and analysis will be combined with regular safety lab measurements during standard routine visits, therefore causing limited additional burden for the patients. The flowchart consisting of the schedule of assessments can be found in **Supplemental Table 1**.

#### 2.2.1 Intervention and control group

For patients in arm A (TDM group) plasma trough concentrations of alectinib will be analyzed directly from the PK samples. Results will be available within 1-2 weeks. In case of C_min,SS_ below the threshold of 435 ng/mL (in arm A) and no or limited toxicity, the patient receives a dose increase with 150 mg BID to a maximum of 900 mg BID. The dose levels are shown in **Table 1**. Four weeks after a dose increase, an extra PK sample is drawn to check whether the intervention was successful, i.e. when TDM target is reached with acceptable tolerability. When patients have adequate exposure (C_min,SS_ ≥ 435 ng/mL), the dose will be continued given no or acceptable toxicity. For patients in arm B, PK samples will be drawn conform the same schedule as patients in arm A. However, these samples will remain blinded to the patients and the treating physician until the end of the study. In case of unmanageable toxicity in both arms (i.e. unbearable or persistent grade 2 toxicity, or grade 3/4 toxicity according to Common Terminology Criteria for Adverse Events (CTCAE) v5.0), the alectinib dose can be reduced by one dose level (150 mg BID) in accordance with standard care unto a minimum of 300 mg BID. Temporary dose interruptions are allowed. If the side effects subside or are no longer present, it is allowed to increase the dose again. To estimate the C_min,SS_ of alectinib, the time of blood draw will be recorded and all patients will keep a paper diary to collect date and time of last intake of alectinib. Alectinib C_min,SS_ will be calculated with the following formula:

**Table 1:** dose levels of alectinib.

Dose level	Alectinib Dose
-2	300 mg BID
-1	450 mg BID
0	600 mg BID
+1	750 mg BID
+2	900 mg BID


$$C_{min,SS}=\;C_{measured}*0.5^{\frac{dosing\;interval-TAD}{t_{1/2}}}$$

Where C_min,SS_ is the estimated minimum plasma concentration, C_measured_ the measured plasma concentration, the dosing interval is the time between two subsequent administrations, TAD is time after dose and t_1/2_ is the elimination half-life of the drug (i.e. 32 hours for alectinib). PK samples should be drawn after at least 4 hours after the last intake of alectinib (after T_max_) (16). It is assumed that this algorithm should perform equivalently, and most likely even better, as imatinib validated by Wang et al, since the half-life of alectinib is longer (16).

### 2.3 Study population

A total of 196 patients will be included in the study, who will all give their written informed consent prior to performing any study related procedures. Approximately 80 patients with ALK+, stage IV NSCLC are diagnosed in the Netherlands per year (5, 17). Almost all these patients will be treated in one of the seven initial participating centers, since there is consensus in the Netherlands to refer patients with rare mutations to a center of expertise, i.e. these seven centers. The inclusion rate is expected to be 65% based on our in- and exclusion criteria. After initiation in the Netherlands, the study will also be launched in other European medical centers, starting with Gustave Roussy (Villejuif, France). This will increase the patient population and speed up the inclusion process. Therefore, it is expected the study fulfilment will be reached in 4 years of inclusion.

#### 2.3.1 Eligibility criteria

The study population will consist of adult patients with locally advanced or metastatic ALK+ NSCLC [stage IIIb to IV by AJCC 8^th^ (18)], starting treatment with alectinib at the standard dose of 600 mg BID in first or second line of care. The ALK fusion must be determined by an EMA-approved test, e.g. RNA sequencing. Patients can either be chemotherapy-naïve or have received one line of platinum-based chemotherapy. Local radiotherapy to reduce pain, as well as asymptomatic and clinically stable (≥ 2 weeks, without steroid treatment) brain or leptomeningeal metastases are allowed.

Main exclusion criteria are significant concomitant diseases or conditions that are potentially aggravated by the treatment, and use of treatments that could interfere with the alectinib therapy. For the complete list of detailed eligibility criteria, see **Supplemental Table 2**.

#### 2.3.2 Sample size calculation

It is expected that 48% of the patients treated with alectinib in our study will be underdosed (i.e. C_min_ < 435 ng/mL at a certain timepoint) based on the dataset of Groenland et al. (13). The observed mPFS for these patients was 12.6 months (95% confidence interval (CI): 9.2 months – not yet reached), which will serve as the assumed median in the subgroup with low exposure in the fixed-dosing group (arm B). The mPFS in the patients with C_min_ ≥ 435 ng/mL had not yet been reached (95% CI: 19.8 months – not yet reached) at the time of analysis, but the HR for the group with C_min_ < 435 ng/mL versus C_min_ ≥ 435 ng/mL was estimated as 2.5 (13). We expect that TDM-guided dosing can improve PFS in 67% of patients with a low exposure, to the extent that they obtain a similar outcome as patients with C_min_ ≥ 435 ng/mL. In this group, a HR (fixed versus TDM-guided dosing) of 2.5 is assumed. In the remaining 33% of patients, TDM-guided dose increases are expected not to be feasible due to toxicity or treatment discontinuation as a consequence of progressive disease (PD), so a HR equal to one is assumed for this subgroup. Altogether, this would result in an expected HR (fixed versus TDM-guided) of 2.0. With 67 events, a power of 80% will be obtained to detect this HR of 2 using the log-rank test and assuming a two-sided significance level of 5%. This translates into an improvement in mPFS from 12.6 months without using TDM-guided dosing to 25.2 months using TDM-guided dosing. Assuming an uniform patient accrual during 48 months and 12 months follow-up after inclusion of the last patient (total study duration of 60 months), 94 patients (47 per group) with C_min _< 435 ng/mL will need to be included for the primary analysis. Since 48% of the patients in the study are expected to have C_min _< 435 ng/mL at a certain timepoint, 196 patients will need to be recruited (13). Dropouts are expected to be kept to a minimum and it does occur, it is expected to be in the first 12 weeks, when substitution is still allowed. Dropouts are therefore not taken into account.

#### 2.3.3 Primary outcome

The primary outcome, based on our aim to improve efficacy of alectinib treatment, is mPFS in the TDM-guided dosing arm for the C_min,SS _< 435 ng/mL subgroup, compared to this subgroup in the fixed dosing arm (standard care). PFS will be calculated as time from start of treatment to PD, death of any cause, whichever occurs first. Patients who do not progress, die, or are lost to follow-up, will be censored at their last available date. PD is defined according RECIST v1.1 and will be based on assessment using CT scans (thorax/abdomen) and MRI of the brain. In case of oligoprogression alectinib may be continued conform standard of care, but the date of oligoprogression (i.e. PD) will be used in PFS calculation. The CT scans will be performed every 8 weeks in the 1^st^ year and every 12 weeks from the 2^nd^ year onwards. The MRI of the brain will be performed every 16 weeks in the 1^st^ year if there is presence of asymptomatic brain metastases at screening and every 24 weeks from the 2^nd^ year onwards. If no brain metastases are found at screening, an MRI of the brain will be performed every 12 months (**Supplemental Table 1**).

#### 2.3.4 Secondary outcomes

There are ten secondary endpoints, defined as follows: 1) Feasibility and safety of TDM, measured as percentage of successful TDM interventions, in which successful is defined as target attainment with manageable toxicity; 2) ORR, defined as partial response or complete response (according to RECIST v1.1) percentage of the total treated population; 3) mOS, where OS is defined as time from randomization to death from any cause in the total population. Patients who do not die or are lost to follow-up will be censored at their last available date; 4) Intracranial PFS, defined as time from start of treatment to PD in the brain or death from any cause, whichever comes first. Patients not experiencing any of these events will be censored at their last available date; 5) Physician adherence to TDM advice, measured as the percentage of dose recommendations that are implemented by the treating physicians; 6) Patients’ medication adherence, estimated by pill counts of returned medication as well as a patient diary on drug intake; 7) Toxicity related to the plasma concentration and dose increases, defined as adverse events (AEs) in the subgroup with C_min,SS _< 435 ng/mL and all C_min,SS_ ≥ 435 ng/mL, and in patients who did and who did not receive a PK-guided dose increase; 8) QoL; this will be determined using the EORTC QLQ_LC13 as addition to the QLQ-C30 questionnaire, and the EQ-5D-5L questionnaire. 9) Cost-effectiveness, this will be determined from both a healthcare and a societal perspective. Costs associated with healthcare resource use in combination with Quality adjusted-life years will be used to determine an incremental cost-effectiveness ratio (ICER); 10) Alectinib-M4 concentrations, which will be measured in the alectinib plasma samples. For the endpoints regarding ORR, mOS, intracranial PFS, the comparison will be made between arm A and B, among patients who had a C_min,SS _< 435 ng/mL at any point during treatment. Feasibility and safety of TDM and physician adherence to TDM advice will only be evaluated in arm A. Patients medication adherence, toxicity, QoL, cost-effectiveness and alectinib-M4 concentrations will be evaluated in all patients.

#### 2.3.5 Exploratory endpoints

Some optional endpoints in the treatment of patients with ALK+ NSCLC are defined: 1) Predictive value of response to treatment (i.e. PFS) by next-generation sequencing (NGS) using AVENIO ctDNA test, with emphasis on acquired resistance mechanisms by the following subgroup of response: a) ctDNA profiles associated with primary resistance PD within 6 months); b) ctDNA profiles associated with acquired resistance (PD 6-24 months); c) ctDNA profiles associated with continuing response of 2 years and more; 2) Concordance of molecular profile between tumor biopsy NGS and ctDNA at baseline. As well as at PD, after performing a re-biopsy as standard of care; 3) Correlation with average daily exposure and reduction in ctDNA levels. To obtain DNA from cell free plasma, blood will be withdrawn at baseline in three Streck^®^ tubes (Streck Inc, Omaha, NE, USA) and one EDTA tube (Vacutainer) to process the buffy coat (consisting of peripheral blood mononuclear cells), to identify tumor-specific mutations using NGS analysis and exclude variants associated with germ-line, clonal hematopoiesis and technical artefacts. Each subsequent cycle, two Streck tubes will be collected. CtDNA will be analyzed using the Avenio ctDNA Expanded covering 77 clinical-relevant markers (19). There is ample experience using this Avenio ctDNA Expanded Kit of large plasma samples (20, 21).

Furthermore, additional data regarding the effects of alectinib on weight gain will be collected. These exploratory endpoints are defined as follows: 4) Parameters such as body weight, body surface area and smoking; 5) Changes in body composition measured on CT-thorax/abdomen, including subcutaneous and visceral adipose tissue measurements, using specialized software; 6) Measurements of appetite and satiety hormones and their relationship to alectinib therapy related weight gain.

### 2.4 Data analysis

Survival endpoints will be analyzed using Kaplan-Meier curves and differences between groups will be tested using the stratified log rank test. The HR and its corresponding 95% CI from a stratified Cox model will be estimated. The median survival will be presented by arm along with 95% confidence intervals using the Brookmeyer and Crowley method. A p-value < 0.05 will be deemed statistically significant.

The effect of biomarkers in the secondary and exploratory study parameters (such as sequencing profile by AVENIO of biopsy) will be explored by correlating the changes in a marker to any outcome (such as OS). This will be performed by crosstabs, t-tests, Mann-Whitney U tests, and Spearman’s rank correlations, depending on the distribution of the data.

ORR will be calculated by arm with exact 95% CI. For comparing response rates in the two-treatment arms accounting for randomization stratification factors, the Cochran-Mantel-Haenszel test will be used at a two-sided 5% level of significance.

For treatment-emergent AEs (i.e., those starting or worsening during the period on treatment) summary tables will be provided by treatment group. Worst-grade AEs will be summarized by system organ class and preferred term, severity (by CTCAE v5.0 criteria) and relation to study treatment. Serious adverse events will be listed. Statistical analyses will be specified in the Statistical Analysis Plan.

Comparative cost-effectiveness analysis will be performed using a health-state transition model, to compare costs and effectiveness between the group treated with TDM-guided dosing and the fixed dosing group. The comparative cost-effectiveness analysis will be performed from both a healthcare and societal perspective, with a lifetime horizon, according to the Dutch guidelines for cost-effectiveness analyses (22).

### 2.5 Study logistics

#### 2.5.1 Duration and termination of the study

It is expected that inclusion of 196 patients will be fulfilled within 4 years. The study is open for enrolment since March 23, 2022 in the first study site. Patients that withdraw from the study within the first 12 weeks may be replaced by other patients. No foreseen risks have been identified that could lead to premature termination of this study. In case of slow accrual, the inclusion period can be extended with one year. With a minimum follow-up of 12 months, the maximum study duration will be 6 years after which analyses will take place.

#### 2.5.2 Safety assessments

The AEs will be assessed every visit and continuously during the study as reported by the patient in between visits. The AEs will be recorded in the electronic Case Report Forms (eCRF) according to the CTCAE version 5.0.

#### 2.5.3 Data management and study monitoring

Study data will be collected and managed using REDCap electronic data capture tools (23, 24). Eligibility and efficacy parameters, compliance to treatment schedules and parameters necessary to evaluate the study endpoints will be documented in the eCRFs. Data collected in the eCRF are derived from the protocol and collected by the investigators of the study. A clinical research monitor will supervise the data entry and checks whether the study is conducted according to the protocol. Monitoring will take place centrally from the sponsor University Medical Center Groningen and annual monitor visits will take place at the study sites. An independent Data Safety Monitor Board (DSMB), will be installed to monitor all cumulative safety data, accrual, proportion of patients with an alectinib C_min,SS_ < 435 ng/mL and treatment exposure, once a year. The DSMB will recommend the principal investigator to continue the trial as planned, to stop or extend recruitment, to extend follow-up or the DSMB will propose protocol changes.

#### 2.5.4 Patient and public involvement

The ADAPT ALEC trial protocol and patient information folder were developed in collaboration with the Dutch patient federation for lung cancer patients ‘Stichting Longkanker Nederland’. They evaluated patient needs and recommended to add QoL in this trial as an outcome parameter. The patient federations ‘Longkanker Nederland’ and ‘ALK Positive Inc and Europe’ fully support this trial and strategies to personalize the treatment on patient-level to improve treatment outcomes.

## 3 Discussion

The purpose of ADAPT ALEC is to investigate in an RCT whether patients with ALK+ NSCLC would achieve better treatment outcomes when using personalized dosing.

The proposed TDM target of 435 ng/mL, was based on the dose-finding study, where patients with exposure in the lowest tertile had less reduction in tumor size compared with the upper two tertiles (C_min,SS_ ≥ 435 ng/mL) (12, 13). The manufacturer of alectinib identified baseline tumor size as only significant covariate for best overall response, while combined average concentration of alectinib and its metabolite M4 was not significantly correlated with best overall response (12). In research of Morcos et al. an exposure-response analysis of alectinib was performed in 207 crizotinib- resistant patients with ALK+ NSCLC. No significant relationship was found between OS and trough levels (14). Given the observational nature of these findings in exposure-response analyses, which are not consistent with each other, a prospective study is necessary to evaluate the hypothesis that TDM of alectinib would result in better treatment outcomes.

Notably, this type of research has not been performed often, and when it was designed it was not always proven successful, as illustrated by the trial of Gotta et al. and the aborted SARC019 trial which tested TDM of kinase inhibitors (25, 26). Gotta et al. provided suggestions to consider when designing such RCTs in the future, which we took into consideration when designing this trial protocol. One of the suggestions was differentiating between efficacy and safety outcomes, which is captured by a primary endpoint based on efficacy, and a secondary endpoint on toxicity (in relation to plasma trough levels of alectinib). Additionally, investigating reasons for prescribers’ non-adherence to dosage recommendations is captured by formulating the physician adherence as a secondary endpoint. The authors also recommended restricting the inclusion to treatment naïve patients. In the ADAPT ALEC protocol, stratification will be applied in randomization for first- and second-line treatment. Another issue raised was that TDM could encourage drug discontinuation in patients intolerant despite acceptable plasma trough concentrations. In this protocol, this is not specifically defined, in case of intolerance it is allowed to reduce the dose, which will happen regardless of plasma levels, before switching therapy, which is in accordance with regular care.

Furthermore, Buclin et al. stated in their structured approach to implement TDM, that cost-effectiveness is likely to be confirmed, given the high prices of targeted anti-cancer agents (i.e. alectinib) and TDM requires only a minor improvement in efficacy to be cost-effective (27). However, evidence for the cost-effectiveness of TDM of targeted anticancer agents is lacking. Therefore, QoL and cost-effectiveness are included in the ADAPT ALEC protocol, which is an important strength of this study design and will help decision making in terms of implementation of TDM. We expect a longer PFS when comparing the subpopulations with low exposure (C_min,SS_ < 435 ng/mL) in TDM and fixed dosing group, while we expect the QoL to be the same in both groups. It is hypothesized that this will result in acceptable costs for gain in life years without being at expense of QoL. In clinical practice (not in this trial), TDM can also lead to a decreased dose in case of a high exposure. This will be captured in a hypothetical scenario, based on data in the trial (decreased dose in case of toxicities) and expert opinion. Taken this into account, it could mean that the incremental cost-effectiveness ratio will be more favorable.

Besides secondary endpoints on efficacy and safety, other valuable data will be collected, for instance alectinib-M4 concentrations. Alectinib-M4 is the major active metabolite of alectinib and is formed by cytochrome P450 3A4 (CYP3A4), which accounts for approximately 40% of alectinib’s metabolism (12). Alectinib and its metabolite M4 are equally active. In case of using CYP3A4 modulators, the ratio of alectinib and M4 concentrations will change without a meaningful change in combined exposure of alectinib and M4. Therefore, in current clinical practice no dose adaptations are recommended in case of concomitant administration of CYP3A4 modulators. When performing TDM of only alectinib without measuring M4, caution is needed that combined exposure is under- or overestimated. The effect of CYP3A4 modulators is not studied in this trial, but alectinib-M4 exposure relative to alectinib plasma concentrations will be an important component to explore as limited data regarding alectinib-M4 exposure is available.

In addition, we included exploratory outcomes, a separate consent is needed for each outcome. First, the use of ctDNA measured in plasma is promising in clinical decision-making of patients with NSCLC through its use in predictive testing, detection of resistance mechanisms and monitoring response to therapy (28). In EGFR mutated NSCLC, clearance of ctDNA was found to be predictive of both PFS and OS (28). Nevertheless, prospective data is lacking and there is a need for larger prospective trials collecting ctDNA of specific mutations and TKIs to be able to use this biomarker (28). The ADAPT ALEC trial aims to aid in this demand to prospectively collect ctDNA in patients with ALK+ NSCLC and assess its predictive value of response to alectinib treatment. Considering the long-term survival achieved with alectinib therapy, longitudinal low-grade toxicity becomes more important. Second, a relatively unexplored adverse effect is weight gain, reported in approximately 10% of patients in the pivotal ALEX trial (8). Currently, the etiology and sequelae of alectinib-induced weight gain remain to be elucidated. In this trial, we aim to unravel this phenomenon by prospective analysis of body composition using CT-images made in regular care, in combination with collection of blood samples for measurement of a broad spectrum of satiety and appetite hormones.

Potential limitation of the study design is the relative short follow-up time of 12 months to demonstrate PFS and no marge for ‘non-evaluable’ patients is included in the sample size calculation. The chance that a patient drops out is considered highest in the first 12 weeks after initiation of treatment, when the protocol allows replacement for a new patient. During the study, accrual and event rate will be evaluated. Potential changes in the number of patients to be recruited and/or in duration of follow-up as a consequence of these evaluations will be discussed with the DSMB.

Another limitation is the fact that this study protocol is based on European standard dosing, while in Japan 300 mg BID is used (29). One hypothesis is that characteristics in the Japanese population (lower body weight and different metabolism) result in relatively higher levels and that they therefore have comparable exposure at a lower dose (30, 31). We, therefore, expect that dosing based on levels in Japan will also be applicable. Patients will start from a different starting dose, in order to have levels in the same therapeutic window as the non-Japanese population.

The expected HR of 2.0 might be slightly challenging, with the risk of ending up with a negative study. With a less extreme HR we would have needed more events and the feasibility of the study would be compromised. The HR of 2.0 is a compromise between a realistic but clinically relevant difference and the feasibility of the study. We therefore believe that the HR is achievable and will not compromise the study.

It is expected that patients will be willing to participate, since the additional burden for patients is minimal. Patients in arm B will not receive a dose increase in case of low exposure, which could be considered unethical as plasma (trough) concentrations are measured but not acted upon. However, arm B represents the patients treated according to current guidelines and thus receiving standard care which is acceptable. Furthermore, many patients are recruited from which only a fraction will be used in the analysis of the primary endpoint and some secondary endpoints, because in forehand it is unknown whether patients will have low or high alectinib exposure. We accept this as the additional burden for all patients is minimal and the obtained data of all patients will provide high quality insights for our secondary endpoints.

Comparing TDM-guided dosing versus standard dosing in a randomized clinical trial setting is the ultimate way to investigate the clinical relevance of practicing TDM of alectinib. The ADAPT ALEC trial has the potential to provide high quality evidence for deciding whether TDM should be implemented as standard of care in alectinib therapy.

## Ethics statement

Ethical approval was given by the Medical Ethical Committee of the University Medical Center Groningen. The trial is registered in the Netherlands trial registry NL9411 (http://www.trialregister.nl/trialreg/index.asp) and in European drug regulatory affairs Clinical Trials EudraCT: 2020-001737-13 (https://eudract.ema.europa.eu). Independently of the outcome, study results will be presented at (inter) national scientific meetings and published as an article in a peer-reviewed journal. All trial documents and results will be stored for 25 years.

## Author contributions

MM and BM wrote the manuscript. MM, BM, NS, AH and AW designed the study protocol. ML-Y performed the sample size calculation and supported the statistical design. VR designed the cost-effectiveness analysis. BM, AW, ESc and WT were responsible for the exploratory endpoint regarding ctDNA. BS, RM and AD were responsible for exploratory endpoint regarding body composition. All authors contributed to the article and approved the submitted version.
