# Supplementary material for: Therapeutic drug monitoring guided dosing versus standard dosing of alectinib in advanced ALK positive non-small cell lung cancer patients: Study protocol for an international, multicenter phase IV randomized controlled trial (ADAPT ALEC)
Source: Front Oncol. 2023 Mar 9;13:1136221. doi: 10.3389/fonc.2023.1136221 (PMC10035072; doi:10.3389/fonc.2023.1136221)
Supplement: Supplementary file 1 [file Table_1.pdf]

Supplementary Material

Therapeutic drug monitoring guided dosing versus standard dosing of alectinib in advanced ALK positive non-small cell lung cancer patients: study protocol for an international, multicenter phase IV randomized controlled trial (ADAPT ALEC)

Marinda Meertens[1] †, M. Benthe Muntinghe-Wagenaar[2] †, Barend J. Sikkema[3], Marta Lopez-Yurda[4], Valesca P. Retèl[5], Marthe S. Paats[6], Rob ter Heine[7], Ed Schuuring[8], Wim Timens[8], Daan J. Touw[9], Job F.M. van Boven[9], Adrianus. J. de Langen[10], Sayed M.S. Hashemi[11], Lizza E.L Hendriks[12], Sander Croes[13], Michel M. van den Heuvel[14], Anne-Marie C. Dingemans[6], Ron H. J. Mathijssen[3], Egbert F. Smit[15], Alwin D.R. Huitema[1,16,17], Neeltje Steeghs[18]‡, Anthonie J. van der Wekken[2] ‡*

† These authors contributed equally to this work and share first authorship
‡ These authors contributed equally to this work and share last authorship

*** Correspondence:**
Corresponding Author: a.j.van.der.wekken@umcg.nl

[1] *Department of Pharmacy and Pharmacology, Netherlands Cancer Institute, Amsterdam, The Netherlands*
[2] *Department of Pulmonology and Tuberculosis, University of Groningen, University Medical Center Groningen, Groningen, The Netherlands*
[3] *Department of Medical Oncology, Erasmus MC Cancer Institute, University Medical Center Rotterdam, Rotterdam, The Netherlands*
[4] *Department of Biometrics, The Netherlands Cancer Institute, Amsterdam, The Netherlands*
[5] *Division of Psychosocial Research and Epidemiology, Netherlands Cancer Institute, Amsterdam, The Netherlands*
[6] *Department of Pulmonary Medicine, Erasmus MC Cancer Institute, University Medical Center Rotterdam, Rotterdam, The Netherlands*
[7] *Department of Pharmacy, Radboud Institute for Health Sciences, Radboud University Medical Center Nijmegen, Nijmegen, The Netherlands.*
[8] *Department of Pathology and Medical Biology, University Medical Center Groningen, University of Groningen, Groningen, The Netherlands*
[9] *Department of Clinical Pharmacy and Pharmacology, University Medical Center Groningen, University of Groningen, Groningen, The Netherlands*
[10] *Department of Thoracic Oncology, Netherlands Cancer Institute-Antoni van Leeuwenhoek Hospital, Amsterdam, The Netherlands*
[11] *Department of Pulmonary Medicine, Amsterdam UMC, VU University Medical Center, Amsterdam, The Netherlands*
[12] *Department of Respiratory Medicine, Maastricht University Medical Center, GROW School for Oncology and Reproduction, Maastricht, the Netherlands*
[13] *Department of Clinical Pharmacy and Toxicology, Maastricht University Medical Center, CARIM School for Cardiovascular disease, Maastricht, the Netherlands*
[14] *Department of Pulmonary Diseases, Radboud University Medical Center, Nijmegen, The Netherlands*
[15] *Department of Pulmonology, Leiden University Medical Center, Leiden, the Netherlands*
[16] *Department of Clinical Pharmacy, University Medical Center Utrecht, Utrecht, The Netherlands*
[17] *Department of Pharmacology, Princess Máxima Center for Pediatric Oncology, Utrecht, The Netherlands*
[18] *Department of Clinical Pharmacology, Division of Medical Oncology, The Netherlands Cancer Institute, Antoni van Leeuwenhoek Hospital, Amsterdam, the Netherlands.*

Supplemental Table 1: flowchart schedule of assessments

**Table 1.1 Screening assessments and procedures**		
**Procedure**	**Screening Visit**	**Within 28 days prior to first dose**
**ELIGIBILITY ASSESSMENTS**		
Informed Consent	X	
Inclusion / Exclusion Criteria	X	
Medical History	X	
**SAFETY ASSESSMENTS**		
Physical Measurements / Physical Examination	X	
ECOG Performance status	X	
Vital Signs (RR and heart rate) and Oxygen Saturation	X	
Length and weight	X	
Assessments of Signs and Symptoms	X	Within 14 days
Concomitant Medication	X	Within 14 days
Chemistry & Haematology tests	X	Within 14 days
ECG (12 lead)	X	
**EFFICACY ASSESSMENTS**		
Radiographic Tumour Assessment (PET/CT-chest/abdomen and CT or MRI brain*)	X	
**BIOMARKER / OTHER ASSESSMENTS**		
QoL questionnaires	X	
Sufficient archived Tumour Tissue or Recent Tumour Biopsy or tumour cell block suitable for NGS	X#	
10 mL EDTA blood for PBMC collection	X#	
30 mL Blood Streck tubes for ctDNA	X#	
12 mL Clot Acivated Tubes and 6mL EDTA for hormonal blood tests (fasting blood draw)	X@	

* Brain metastases occur frequently in NSCLC patients with an ALK fusion. In case of proven brain metastases these have to be followed up by MRI (preferably, or CT when indicated). [#]optional for those centers who participate in collecting ctDNA [@]optional for those centers who participate in collecting appetite and satiety hormones

Table 1.2 Study assessments and procedures

**Procedure (Cycle 1 is 28 days; cycle 2-7 is 56 days; cycle 8 etc. is 84 days**)**	**C1D1**	**C2D1** Wk 4	**C3D1** Wk12	**C4D1** Wk 20	**C5D1** Wk 28	**C6D1** W36	**C7D1** Wk44	**C8D1** Wk 52	**C9D1** Wk 64	**C10D1** Wk 76	**Subsequent cycle - D1**	**Treatment discontinuation**
**SAFETY ASSESSMENT**												
Physical Measurements & ECOG Performance Status	X	X	X	X	X	X	X	X	X	X	X	X
Vital Signs/weight	X	X	X	X	X	X	X	X	X	X	X	X
ECG	X	X										
Concomitant Medication	X	X	X	X	X	X	X	X	X	X	X	X
Pharmacokinetic sample (Alectinib level 4 mL EDTA)∞ [Ω]		X	X	X	X	X	X	X	X	X	X	X
Chemistry & Haematology tests	≤14 days	X	X	X	X	X	X	X	X	X	X	X
Adverse Events Assessments	***Continuously during the study***											
**EFFICACY ASSESSMENT**												
Diagnostic CT-thorax/abdomen		X[$]		X[$]	X[$]	X[$]	X[$]	X[$]	X[$]	X[$]	X[$]	X[$]
MRI brain				X[‡$]		X[‡$]		X[$]		X[‡$]	X[‡$]	
**BIOMARKER / OTHER ASSESSMENTS**												
Quality of Life questionnaires			X		X		X	X	X	X	X	X
2x10mL blood Streck tubes - ctDNA	≤14 days[#]	X[#]	X[#]	X[#]	X[#]	X[#]	X[#]	X[#]	X[#]	X[#]	X[#]	X[#]
2x 6mL Clot Activation Tube & 1x 6mL EDTA tube – hormones[@]			X[@]					X[@]				
**CLINICAL DRUG SUPPLY**												
Cohort A: Alectinib 600mg BID	X	X	X	X	X	X	X	X	X	X	X	
TDM based drug adaptation		X	X	X	X	X	X	X	X	X	X	
Cohort B: Alectinib 600mg BID	X	X	X	X	X	X	X	X	X	X	X	

** After dose increase because of alectinib $C_{min,SS}$ <435 ng/mL, cycle is 28 days. [∞] >4 hours after last dose. [Ω] In case of short-term use of CYP3A4 modulating agent: measuring plasma concentration >14 days after last dose administration. [$] Within a window of +/- 7 days. [‡]Every other cycle if brain metastases at baseline (i.e. every 16 weeks during the first year, every 24 weeks from the second year onwards, thus every even cycle), otherwise every 12 months [#]optional for those centers participating in collecting ctDNA [@]optional for those centers who participate in collecting appetite and satiety hormones. *C: Cycle. D: Day. Wk: Week.*

Supplemental Table 2: In- and exclusion criteria

**Inclusion criteria**	**Exclusion criteria**
1. Patients with locally advanced or metastatic NSCLC (stage IIIB to stage IV by AJCC 8th)<br><br>2. Male or female ≥18 years old<br><br>3. ECOG Performance Status of 0−4<br><br>4. Histologically or cytology confirmed NSCLC<br><br>5. Documented ALK rearrangement based on an EMA approved test<br><br>6. Patients can either be chemotherapy-naïve or have received one line of platinum-based chemotherapy<br><br>7. Patients with brain or leptomeningeal metastases are allowed on the study if the lesions are asymptomatic without neurological signs and clinically stable for at least 2 weeks without steroid treatment. Patients who do not meet these criteria are not eligible for the study<br><br>8. Measurable disease (by RECIST criteria version 1.1) prior to the first dose of study treatment<br><br>9. Signed written Institutional Review Board (IRB)/Ethical Committee (EC) approved informed consent form, prior to performing any study-related procedures<br><br>10. Observational other studies are allowed for patients included in this study<br><br>11. Local radiotherapy is allowed for pain	1. Any significant concomitant disease determined by the investigator to be potentially aggravated by the investigational drug<br><br>2. Consumption of agents which modulate CYP3A4 or agents with potential QT prolonging effects within 14 days prior to admission and during the study (see concomitant medication restrictions)<br><br>3. Any clinically significant concomitant disease or condition that could interfere with, or for which the treatment might interfere with, the conduct of the study, or absorption of oral medications, or that would, in the opinion of the Principal Investigator, pose an unacceptable risk to the subject in this study<br><br>4. Any psychological, familial, sociological or geographical condition potentially hampering compliance with the study protocol requirements and/or follow-up procedures; those conditions should be discussed with the patient before trial entry
